# Peptone Supplementation of Culture Medium Has Variable Effects on the Productivity of CHO Cells

**Published:** 2014

**Authors:** Fatemeh Davami, Lucia Baldi, Yashas Rajendra, Florian M. Wurm

**Affiliations:** 1 Laboratory of Cellular Biotechnology, Ècole Polytechnique Fédérale de Lausanne, Switzerland; 2 Biotechnology Research Center, Pasteur Institute of Iran (IPI), Tehran, Iran

**Keywords:** Feeding strategy, mammalian cell culture, plant peptones, recombinant proteins, stable CHO cell lines

## Abstract

The optimization of cell culture conditions for growth and productivity of recombinant Chinese hamster ovary (CHO) cells is a critical step in biopharmaceutical manufacturing. In the present study, the effects of the timing and amount of peptone feeding of a recombinant CHO cell line grown in a basal medium in serum-free suspension culture were determined for eight peptones of different origin (plant and casein). The amino acid content and the average molecular weight of the peptones chosen were available. In optimized feeding strategies with single peptones, increase 100 % volumetric productivity and 40 % in cell number were achieved. In feeding strategies with two peptones, several combinations stimulated protein productivity more than either peptone alone, depending on the peptone concentration and time of feeding. Some peptones, which did not stimulate productivity when added alone proved to be effective when used in combination. The combined peptones feeding strategies were more effective with peptones of different origin. Our data support the notion that the origin of peptones provides some guidance in identifying the most effective feeding strategies for recombinant CHO cells.

Chinese hamster ovary (CHO) cells are the most widely used mammalian host for the production of recombinant protein biopharmaceuti-cals, as they are proven to be safe, capable of providing the correct protein glycosylation, and adapted to suspension growth at high cell density in large bioreactors. Industrial bioprocesses are now essentially based on serum-free media (SFM) and, more generally, animal component-free media, which have both economical and safety advantages over media containing animal-derived products such as serum ^1^^; ^^2^. Peptones are water-soluble protein hydrolysates of chemically undefined nature, containing peptides, amino acids, and inorganic salts as well as other compounds such as lipids, vitamins and sugars ^3^^; ^^4^. Different studies have shown that plant-derived peptones can improve cell growth and enhance specific productivity (µg protein/cell/day) and volumetric productivity (concentration of protein in conditioned media) in both stable and transient expression systems ^5^^-^^8^. However, the positive effect of peptones is not the same for all cell types and clones and similar feeding strategies may cause different responses in different systems. Consequently, a peptone-supplemented bioprocess needs to be optimized for a specific cell line or clone. In this study, we focused on a few plant-derived and casein-derived peptones with available amino acid profiles. Here, for the first time we compared eight peptones with different amino acid compositions, alone or in two-peptone combina-tions, and measured their effects on productivity on this special antibody-expressing stable CHO cell lines with a new insight to amino acid profile of peptones as feeding strategies of mammalian cell cultures.

## Materials and Methods

**Cells**

Two stable CHO DG44-derived cell lines (clones 1 and 2) expressing a human anti-RhesusD IgG ^9^ were used in this study. Both clones were established following gene delivery with the piggyBac transposon ^10^.

**Media and reagents**

RPMI 1640 medium was purchased from Lonza (Verviers, Belgium). All medium supplements used in this study were purchased from Sigma-Aldrich (St. Louis, MO). A basal medium based on RPMI 1640 (LBTC-CDM; Rajendra and Wurm, unpublished) was developed in this laboratory and supplemented with 44 mM glucose and 6 mM glutamine. Peptones were purchased from Organotechnie (La Courneuve, France). The total amino acid composition, molecular weight distribution, and free amino acid content of the peptones used in this study are provided by the manufacturer and available at the address: http://www.organotechnie.com. Table 1 resumes the available data about peptones origin, total amino acid content and molecular weight distribution. Supplementary informations about individual amino acids content of the peptones are provided as electronic supplementary material in Tables S1 and S2. Peptone stock solutions (20%, w/v) were prepared in ultrapure water, sterilized by filtration through 0.2 µm filters, and stored at 4 ºC.

**Osmolality measurement**

The osmolality of media was measured using a freezing point computerized micro-osmometer (Multi-Osmette™ 2430, Precision Systems, Natick, MA). The osmolality of the peptone-supplemented media was between 310 and 330 mOsm.

**Protein production**

The concentration of a human recombinant IgG in the culture medium was determined by sandwich ELISA as previously described ^11^. The IgG volumetric productivities of the batch cultures were normalized relative to the control without peptone and expressed either in percentage (Fig. 2, 3 and 4) or fold variation (Fig. 5). The absolute values of the control cultures without peptone varied among the different experiments due to differences in the initial cell density and to variation in productivity after repeated subcultivation (table 1).

**Cell cultivation**

Clones 1 and 2 were routinely cultivated in ProCHO5 medium (Lonza, Verviers, Belgium) containing 13.6 mg hypoxanthine/L, 3.9 mg thymidine/L, and 4 mM glutamine. Cultures were agitated at 110 rpm in square-shaped glass bottles (Schott AG, Mainz, Germany) on a ISF-4-W orbital shaker (Kühner AG, Birsfelden, Switzerland) at 37 ºC in a 5 % CO_2_ atmosphere ^12^. Biomass was determined by the packed cell volume (PCV) method using PCV tubes (TPP, Techno Plastic Products AG, Trasadingen, Switzerland) ^13^. A cell density of 1 × 10^6^ cells mL was equivalent to a PCV of 0.25 % for cells under standard cultivation conditions at 37 °C. For each experiment, cells were centrifuged at 1500 rpm for 3 min, resuspended in LBTC-CDM at a cell density of 1.0 × 10^6^ cells mL, and maintained as described above. On the day of peptone addition, the cells were centrifuged and transferred to 5 ml fresh medium (RPMI 1640 or LBTC-CDM) containing peptone(s) in TubeSpin^®^ Bioreactor 50 tubes (TPP). The cultures were agitated at 180 rpm on an ISF-4-W orbital shaker at 37 ºC in a humidified 5 % CO_2_ atmosphere. Cell density and viability were assessed by the trypan blue dye exclusion method using a haemocytometer. The PCV was determined as described above.

**Table 1:** Total amino acids content, average molecular weight (MW) and MW distribution of the peptones evaluated in this study

		***Catalogue n.***	***Origin***	***Name***	***Total amino acid content (g/100 g)***	***Average MW (daltons)***	***MW distribution (%)***			
***Peptone*** a	< 0.3 kd					0.3 - 1 kd	1 -10 kd	> 10 kd
**1**		19544	Casein	Casein Peptone Plus	85.1	491	38.5	53.0	8.5	0
**2**		19559	Wheat	Wheat Peptone E1	78.5	474	18.1	79.4	2.5	0
**3**		19685	Soy	Soy Peptone A3 SC	55.2	227	56.0	41.4	2.6	0
**4**		19516	Casein	Casein Peptone N1	80.5	681	21.6	60.0	18.4	0
**5**		19649	Soy	Soy Peptone A2 SC	53.8	503	30.6	60.8	8.6	0
**6**		19546	Casein	Casein Peptone E1	82.4	840	23.5	48.6	27.8	0.1
**7**		19553	Casein	Tryptone N1	81.6	490	31.7	60.1	8.2	0
**8**		19885	Soy	Soy Peptone E-110	49.4	1’206	31.1	48.7	18.5	1.9

a Data available from Organotechnie (www.organotechnie.com)

**Fig. 1:**
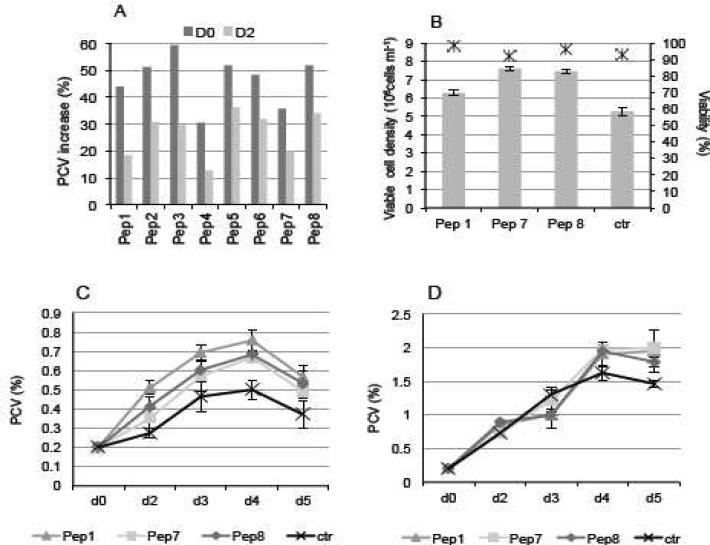
The effect of peptones on cell density, biomass and PCV values in different media.** Panel a**: CHO clone 1 cells were inoculated in RPMI 1640 medium and the different peptones were added at a concentration of 2 g /L on two different times: the day of inoculation (day 0, ■) or 2 days after inoculation (day 2, ■). The relative biomass increase of cultures supplemented with peptones was expressed as a percentage (%) increase to the PCV value of parallel cultures without peptones. Data were measured on day 3 of culture. **Panel b:** Viable cell number (columns) and viability (dots) of clone 1 cultivated in RPMI 1640 medium in the presence of 2 g /L of the indicated peptones, measured on day 3. The indicated peptones were added at the time of cell inoculation.** Panel c:** PCV values (%) of clone 1 cultivated in RPMI 1640 medium in the presence of 2 g /L of the indicated peptones and measured on day 3. The indicated peptones were added at the time of cell inoculation.** Panel** **d:** PCV values (%) of clone 1 cultivated in LBTC-CDM medium in the presence of 2 g /L of the indicated peptones and measured on day 3. The indicated peptones were added at the time of cell inoculation

## Results

**The effect of peptones is media and clone - dependent**

CHO clones 1 and 2 were cultivated in two different chemically defined media (RPMI 1640 and LBTC-CDM) to which individual peptones were added at a concentration of 2 g/L. In the two media, peptone addition had a positive effect on biomass accumulation (not shown). For clone 1, the positive impact of peptone addition on biomass accumulation was more evident than for clone 2. This cell line was therefore chosen for further studies. By day 3 post-inoculation, all peptones tested showed a positive effect on biomass accumulation for clone 1 cultures in RPMI 1640 medium when peptone was added on the day of inoculation (day 0) or on day 2 post-inoculation (Fig. 1a). In addition, the day of inoculation seemed better (Fig. 1a), for the subsequent experiment clone 1 was inoculated in parallel in RPMI 1640 medium, LBTC-CDM medium, and ProCHO5 medium, either in the presence or absence of three randomly chosen peptones (Pep 1, 7 and 8). After 3 days of culture, the viable cell density in LBTC-CDM medium increased in the presence of the three peptones relative to the control culture (Fig. 1b). In contrast, a positive effect of peptone addition was not observed in ProCHO5 medium, which contains plant-derived hydrolysates. Viability on day 3 was above 90 % in LBTC-CDM medium. The addition of peptones to a basal medium (RPMI 1640) stimulated the cell growth more effectively compared to the control in the first 4 days of culture (Fig. 1c), although the overall improvement in biomass was lower in LBTC-CDM (Fig. 1d). RPMI 1640 medium did not sustain growth and productivity of clone 1 on a 7-days process (Fig 1c). Consequently, in all the successive experiments, the effect of peptones on productivity of clone 1 was evaluated in LBTC-CDM.

**Effect of peptone concentration and time of feeding on productivity**

**Fig. 2:**
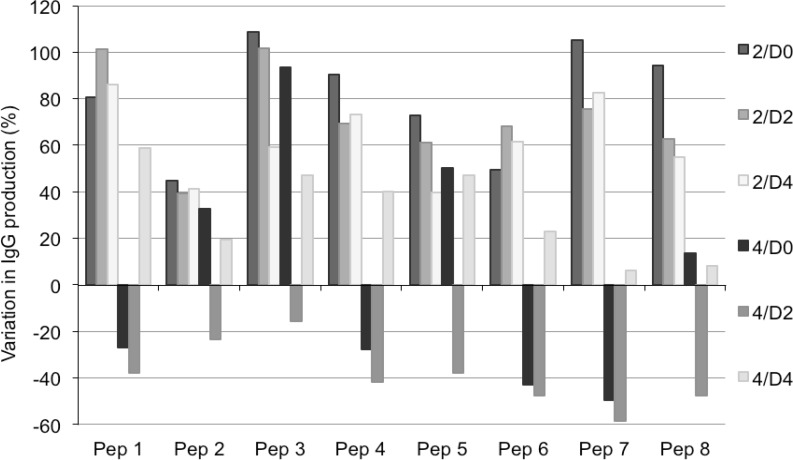
Variation in IgG production levels in the presence of single peptones. CHO clone 1 was cultivated in LBTC-CDM medium in the presence of the indicated peptone at 2 g /L or 4 g /L added on day 0, day 2, or day 4 of culture. Data are expressed as the percentage increase in titers relative to the control culture without peptone. The control cultures produced between 60 and 80 mg IgG /L after 7 days. Cells were inoculated at a density of 1 x 10^6^ cells mL

The effect of peptone feeds at different days (days 0 to 4) after inoculation was tested at different final peptone concentrations (1, 2 and 4 g peptone/L) in LBTC-CDM medium. No significant effect on volumetric productivity of clone 1 was observed with any of the eight peptones tested when fed at a final concentration of 1 g peptone/L compared to titers obtained without peptone feed. At higher concentrations of peptones (2 and 4 g peptone/L), most of the peptones had a positive effect on productivity when added early, either at the time of inoculation (day 0) or on day 2 (Fig. 2). The effects of peptone feeds at 4 g/L on day 0 had a negative impact on final IgG yields for all peptones tested, while later addition (day 4) improved the titers for all the peptones tested (Fig. 2). Similarly to other reported studies with hydrolysates in CHO cells, the most effective concentration to improve productivity was 2 g/L (Fig. 2) ^4^^; ^^5^^; ^^14^ . At a concentration higher than 5 g peptone/L cell growth was inhibited. The addition of peptone on day 6 did not improve IgG titers for any of the peptones tested (data not shown).

The highest impacts on protein yield were observed when feeding with Pep 1 and Pep 3 on day 2, and with Pep 7 and Pep 8 fed on day 0 (Fig. 2). Pep 2 had the least effect on recombinant IgG titers in all single-feed approaches tested (Fig. 2). This wheat-derived peptone has a remarkably higher content of glutamate (29 g/L) and proline (9 g/L) when compared to the other peptones, while the concentration of aspartate was the lowest (2.9 g/L) (see supplementary material Table ST1).

**Effect of peptones in mixed feeding strategy on productivity**

In order to test if the mixing of two peptones could have a synergistic effect in promoting recombinant protein production, 28 two-peptone mixtures at 1:1 (w/w) ratios of each were prepared from the eight different peptones. These mixtures were added to cells on day 0 or day 2 post-inocul-ation at a final concentration of 2 g peptone/L.

**Fig. 3:**
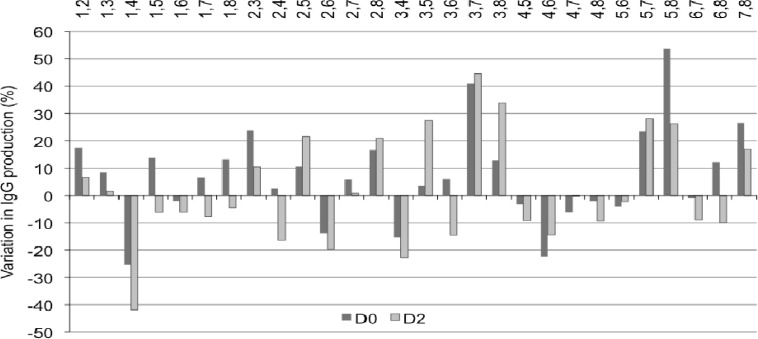
Effect of mixed peptone feeding at 2 g/L on IgG production. CHO clone 1 cells were cultivated in LBTC-CDM medium in the presence of 1:1 (w/w) mixtures of 2 peptones at 1 g/L final concentration of each peptone (total peptone concentration: 2 g/L) added on day 0 (■) or day 2 (■) post-inoculation. Peptones mixes are indicated above the x-axis. Data are expressed as a percentage variation in IgG yield relative to the control culture without peptones. The yield of the control cultures was between 120 and 180 mg IgG/L after 7 days. Cells were inoculated at a density of 1 x 10^6^ cells mL

When added individually, peptones concentration of 1 g/L did not affect productivity for any of the peptones tested (not shown). The addition of 18 of the 28 mixtures at 2 g/L promoted an increase in IgG production (Fig. 3). The other 10 combinations had a negative impact on the final IgG titers, in particular when added on day 2 (Fig. 3). Some mixtures induced an increase of recombinant IgG production when added either on day 0 or on day 2 (Fig. 3). The most effective two-peptone combinations increased titers by up to 40 - 50 %. (Pep 3+7, Pep 3+8, and Pep 5+8). Remarka-bly, all the combinations containing Pep 4 had a negative impact on productivity, both if added on day 0 or on day 2. In comparison with single peptone feeding, the 2-peptone combinations at 2 g/L did not further improve productivity of clone 1 in LBTC-CDM medium, as shown in figures 2 and 3.

**Fig. 4:**
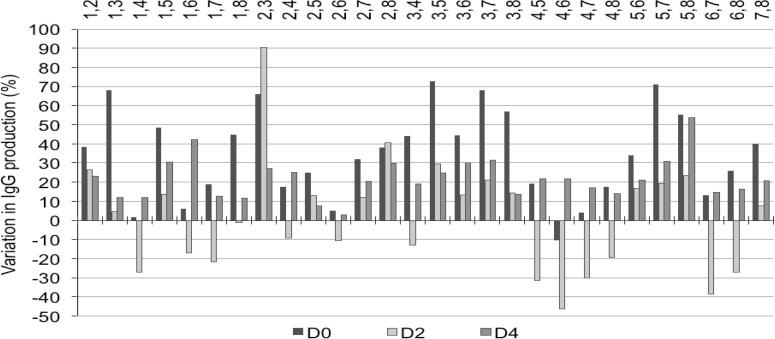
Effect of mixed peptone feeding at 4 g/L on IgG production. To cultures of CHO clone 1 cells in LBTC-CDM medium, mixes of two peptones were added at 2 g/L final concentration of each peptone (total concentration: 4 g peptone/L) on 3 different cultivation days (day 0, day 2, or day 4). Peptones mixes are indicated above the x-axis. The data are expressed as the percentage variation in IgG production relative to the control cultures without peptones. The control yielded between 120 and 180 mg IgG/L after 7 days of culture. Cells were inoculated at a density of 1 x 10^6^ cells mL.

**Fig. 5.**
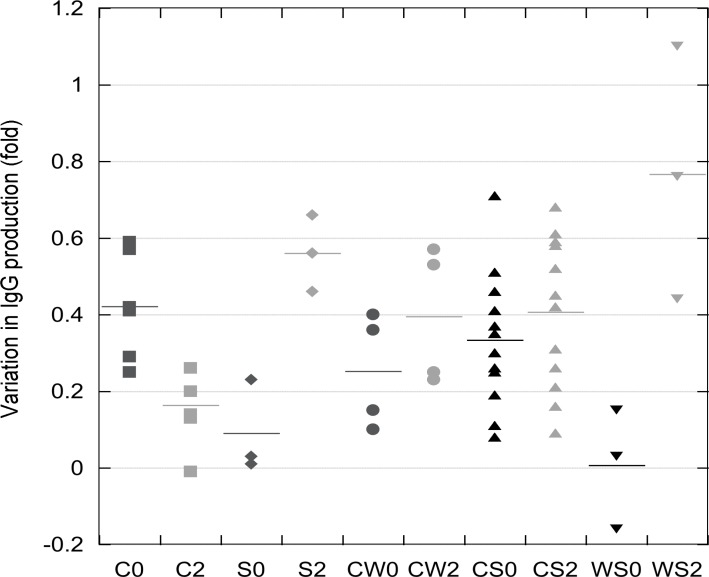
Effect of mixed peptone feeding strategies compared to single peptone feeding strategies.Data on the Y-axis value express the difference in productivity expressed as fold variation over control without peptone, calculated according to the formula: Y = V_(a+b)_ – [(V_a_ + V_b_)/2] where: V_(a+b)_ represents the fold variation in productivity of the 2-peptone mix at 4 g/L (data from Fig. 4); V_a_ and V_b_ represent the fold variation in productivity of the single peptones (a) and (b) added each at 2 g/L (data from Fig.2); Consequently, [(Va + Vb)/2] represents the expected average effect of the two individual peptones. The data obtained were grouped by peptone category and by day of peptone addition post-inoculation (0 = day 0, black dots; 2 = day 2, grey dots). Categories on the X-axis: C, casein + casein; S, soy + soy; CW, casein + wheat; CS, casein + soy; WS, wheat + soy. The numbers refer to the day of peptone addition. A zero value represents no difference in productivity to the expected average effect of single peptone addition

The same 28 two-peptones mixes at 1:1 (w/w) ratios were also used to test the volumetric productivity of clone 1 in LBTC-CDM medium at a final concentration of 4 g/L. In this way, each peptone was added at 2 g/L. Most peptone mixtures enhanced the IgG titers (measured on day 6) when added on day 0 (seeding) (Fig. 4) Peptones with similar amino acid profile (Pep 1, Pep 4, Pep 6, and Pep 7, all from casein) were most effective when mixed with different peptones (Pep 3, Pep 5, and Pep 8, soy bean derived, see Tables 1 and 2). The timing of addition of these peptones was found to be critical. The addition of the mixture was usually most effective during the first day of cultivation (day 0) (Fig. 4). The mixture of two casein-derived peptones had the most negative effect on titers when added on day 2 (see Pep 4+6, Pep 4+7, Pep 1+4, Pep 1+6, Pep 1+7, Pep 6+7), but the later addition (day 4) was beneficial (Fig. 4). Pep 2 (derived from wheat), which showed the most different amino acid composition, improved titers to 65 % and 90 % if added together with Pep 3 on day 0 and day 2, respectively (Fig. 4). The three soy-derived peptones were more effective when fed earlier to the culture. Pep 3 (derived from soy), composed of a large fraction of very small MW peptones (56 % < 0.3 kd, see Table 1), was the most effective in titer improvement at 4 g/L final concentration. The mixture containing soy-derived peptones (Pep 3, 5 and 8) induced titer improvements in almost every combination, and the titers were improved up to 90 % when used in combination with the wheat-derived peptone (Pep 2) (Fig. 4). Soy bean-derived Pep 5 was also very effective in all the two-peptone mixtures at 4 g/L (Fig. 4). The effect of 2-peptone mixtures added on day 0 or day 2 at 4 g/L (data presented in figure 4) was expressed as fold variation over control and compared to the averaged effect of the single individual peptones addition (data reported in Fig. 2). The results were grouped by each category of five peptone mixtures (casein + casein, soy + soy, casein + wheat, soy + casein, soy + wheat) and plotted according to the day of feeding (day 0 or day 2). Fig 5 shows a dot plot of the data obtained. The variation in IgG production obtained with the 2-peptone mixtures were positive in most cases, revealing that the addition of 4 g/L of any two peptones mixture, either of the same origin or not, stimulated protein production more than the average of the individually added peptones in the same concentration (2 g/L).

Soy - derived peptones have been successfully used to enhance protein-free media in batch and fed-batch processes with recombinant CHO cells ^4^^; ^^5^^; ^^14^^; ^^15^. Casein-derived peptones (Pep 1, 4, 6, 7), which present a higher total amino acid content (> 80%, see Tab^l^e 1), were more effective when mixed with soy-derived peptones (Pep 3, 5, 8), which all have lower total amino acid content (around 50%, see Table 1 and Fig. 4). The six mixtures containing peptones derived from casein did not induce an improvement in recombinant protein production (Fig. 4). All the casein-derived peptones have higher total concentration of some amino acids compared to plant-derived peptones (Lys, Thr, Met, Leu, Ile, Val: see supplementary Table ST1). Pep 6 has the highest free tyrosine and histidine content (65.6% and 41.9%, see supplementary Table ST2) compared to all other peptones. Furthermore, Pep 6 has a high average molecular weight with almost 30 % of the total having a size between 1 and 10 kDa, the highest among the casein-derived peptones tested (see Table 1). In two-peptone mixtures, this peptone was efficient only with a soy-derived peptone (Pep 3 or Pep 5, see Fig. 4). The casein-derived peptone Pep 4 showed the lowest beneficial effect, and the titers were systematically reduced when Pep 4 was supplemented in any two-peptones mixture on day 2 of culture. Pep 4 is characterized by the absence of free aspartate, glycine and proline, while the percentage of free cysteine (28.5%) is the highest of all the peptones tested (see supplementary Table ST2). Whether these features have a direct impact on CHO cells productivity remains to be elucidated.

## Discussion

Peptones are widely used as supplement for serum-free culture media. The peptones used in this study were chosen among the commercially available peptones of plant and casein origin whose molecular weight distribution and amino acid content were available fvia the supplier when the study was done. The effects of peptone-mediated improvement of a basal serum-free medium were analyzed by assessing the growth and productivity of a recombinant CHO clone in batch cultures. The results presented in this study demonstrate that the positive effect of peptones on r-protein expression was mainly due to the growth-promoting effect of the peptones, in agreement with previously published data ^4^^; ^^14^. The effect of peptones was influenced by (i) their final concentration and (ii) their time of addition to the culture. The growth-promoting effect was present in the in-house developed chemically defined medium, while in the peptone-rich ProCHO5 medium, no evident positive effect on biomass could be observed. We did not find a major modification in the osmolalities of the peptone-supplemented culture media, with any of the peptone concentrations tested. We tested peptone addition on different days in batch processes. Our data show that for some peptones (Pep 3, 5, 7 and 8) earlier addition was the most effective.

We evaluated the effect on productivity of two-peptone mixes. Only a few recent studies have reported the use of mixed peptones feeding strategies with CHO cells ^6^^; ^^16^. The synergistic effect of combined addition of 4 g /L of two peptone mixtures on day 2 was generally stronger than the addition on day 0, with the exception of casein + casein peptones (Fig. 5). These results suggest that a feeding strategy based on combined peptones of different origin may occasionally occur in a synergistic effect on cell growth and productivity. This synergistic effect observed in some cases could be considered as a consequence of the diverse amino acids composition of the peptones, although the molecular mechanisms of the growth promoting effect of peptones are not

fully understood ^3^^; ^^5^^; ^^17^^; ^^18^.

As shown in table 1, the total amino acid contents of the peptones studied did not exceed about 80% in mass, with soy-derived peptones containing the lowest percentage of amino acids (about 50%). Previous studies revealed that peptone hydrolysates might contain a variable proportion of other nutrients, such as sugars, lipids, vitamins, nucleic acids and minerals ^3^^; ^^19^^; ^^20^. We can therefore suppose that the observed effects may be due at least in part to other nutrients then amino acids possibly present in the hydrolysates.

It has been proposed that peptide molecules may act as growth factors or anti-apoptotic factors, rather than serving as simple feeds. In particular, Lys and Gly-containing synthetic oligopeptides have been shown to promote recombinant protein expression both in CHO cells and hybridomas ^21^^; ^^22^. The hypothesis that is known or yet to be identified components in chemically undefined protein hydrolysates (lipids, hormons, vitamins, salts, etc) may be responsible both for their growth-promoting activity, and for the observed lot-to-lot variations have been recently raised using an NMR-based ^23^ or near-infrared spectroscopy ^24^ analyses of peptones and raw materials. The growth-promoting activity of peptones may have a dual effect in batch cultures, while it may promote rapid cell growth on the first days of culture, it may lead to early depletion of vital nutrients and concomitant release of toxic metabolites, which eventually leads to a more rapid decline of culture viability, apoptosis and release of proteases which may degrade the product. In industrial processes, various strategies aiming to decrease cell growth during the production phase are routinely applied ^25^.

The peptones tested in the present study had all a similar molecular weight distribution, with over 80% of all fractions below 1 kd (see Table 1) and almost 100% below 10 kd. Lower molecular weight fractions of plant peptones have been proven to better support cell growth and prolong cell viability, besides facilitating downstream product recovery ^16^. Pep 2, derived from whey, has the lowest content of Lys, Asp and Trp but the highest content of Glu, Gly and Pro in the form of peptides and not as free amino acid. In a two-peptone feeding strategy, Pep 2 proved to be very efficient, while it had a negative or low impact on productivity when added alone. On the other side, the most efficient peptones (Pep 1, 3, 7, and 8) were not more efficient in the 2-peptones combinations at the concentration tested. Our data suggest that the optimal concentration is likely to vary among the different peptones, and the effect of mixed peptone feeds on productivity cannot be easily predicted by testing the peptones individually. The time of feeding also plays an important role. In the present study, we considered the effect of peptone feeding on volumetric productivity, which may result either from increased specific productivity or from increased biomass, or even a combination of the two effects.

In a recent report, a proteomic approach was applied to identify in CHO cells intracellular protein with either induced or suppressed expression upon peptones feeding ^26^. The expression of several proteins involved in cell proliferation, metabolism and protein folding/ secretion was induced by peptone supplementation, while other proteins involved cell cycle arrest and apoptosis induction were downregulated, suggesting that the growth-promoting effect of peptones acts through multiple molecular targets. The same group executed a design of experiment approach to analyze the effect of ten mixtures of 3 hydrolysates (yeast, soy and wheat) in supporting cell growth and recombinant protein production of two different CHO clones ^6^. Our data are overall in agreement with the findings made by these authors on the following points: (i) wheat hydrolysates were the least effective in promoting productivity of our specific antibody expressig cell clone, and (ii) a synergistic effect on productivity can be obtained by mixing hydrolysates of different origin. Also, a new insight has been included to amino acid profile of peptones as feeding strategies of mammalian cell cultures which would hopefully lead to predictable process development strategies.

The results reported here show that a simple feeding strategy with protein hydrolysates was allowed to improve r-protein titers in a CHO cell clone cultivated in a basal serum-free medium. Furthermore, the two-peptone feeding strategy tested here may give unpredictable results, not always corresponding to the effects of the single peptones fed individually. Peptone concentration and time of addition play a critical role in the feeding strategies. Our high-throughput system, the TubeSpin^®^ bioreactor 50 system based on orbital shaking, proved again to be an efficient tool for cell culture optimization^27^^; ^^28^. The data presented in this study suggest that cell-line tailored feeding strategy based on peptone feeds can be developed which allow improving the productivity of recombinant CHO cell lines in basal media.
